# BVED方案序贯自体造血干细胞移植治疗复发难治侵袭性淋巴瘤合并噬血细胞综合征2例

**DOI:** 10.3760/cma.j.issn.0253-2727.2022.08.016

**Published:** 2022-08

**Authors:** 睿 姜, 益莲 杨, 微 吴, 婧妍 邱, 红岭 秘, 莉 王, 磊 范, 卫 徐, 建勇 李, 华渊 朱

**Affiliations:** 南京医科大学第一附属医院血液科，浦口慢淋中心，南京 210029 Pukou CLL Center, Department of Hematology, the First Affiliated Hospital of Nanjing Medical University, Nanjing 210029, China

例1，女，37岁，2020年3月入院。既往经腹腔淋巴结活检明确诊断为弥漫大B细胞淋巴瘤（DLBCL），以CHOP方案一线治疗5个周期获得部分缓解（PR），复发后以Gemox方案二线治疗2个周期、R-GDP方案治疗5个周期再次获得PR。既往有“慢性乙型肝炎”病史、放射性物质接触史2年。血常规示HGB 60 g/L、PLT 67×10^9^/L；铁蛋白（SF）2 500 µg/L（参考值24～336 µg/L）；乳酸脱氢酶（LDH）984 U/L（参考值120～250 U/L）；可溶性白细胞介素2受体（sCD25）58 917 ng/L（参考值0～2000 ng/L）；骨髓穿刺涂片见噬血细胞；腹腔淋巴结活检免疫组化：CD30（+），CD3（−），CD10（−），CD20（−），Bcl-6（+），MUM1（+），BCL-2>50％，Cyclin D1（−），C-MYC>40％，P53>75％，Ki-67>75％，原位杂交EBER（−）；FISH：BCL-2，C-MYC，P53，BCL-6未见异常；增强CT示腹主动脉旁多发淋巴结肿大（最大66 mm×45 mm）。诊断为“DLBCL（non-GCB）Ⅱ期B组，噬血细胞综合征（HLH）”。2020年3月起予R-BVED方案化疗（利妥昔单抗375 mg/m^2^第1天，苯达莫司汀70～90 mg·m^−2^·d^−1^第2、3天，长春地辛3 mg/m^2^第1天，依托泊苷100 mg·m^−2^·d^−1^第1、4天，地塞米松15 mg/d第1、4天），同时予阿糖胞苷、甲氨蝶呤、地塞米松鞘内注射。首疗程化疗后骨髓抑制期发生发热，予以抗感染、输注血小板和红细胞生成素（EPO）对症治疗后好转。第2周期化疗后出现Ⅲ级中性粒细胞减少和Ⅱ级胆汁酸升高，对症治疗后好转。2个周期化疗后增强CT示腹主动脉旁较大淋巴结较前缩小75％，根据Lugano标准疗效评估达PR，复查SF 991 µg/L，第4周期化疗后复查PET/CT示“腹主动脉旁多发肿大淋巴结，FDG未见增高（Deauville评分1分）”；骨髓未见噬血细胞，SF 434 µg/L，sCD25 1 752 ng/L。2020年6月以大剂量依托泊苷（1.6 g/m^2^）行外周血干细胞动员、采集后继续完成第5、6周期R-BVED方案化疗，复查增强CT示“腹主动脉旁较大淋巴结约23 mm×11 mm，达到完全缓解（CR）”。2020年10月予BEAC方案预处理（卡莫司汀400 mg、−6 d，依托泊苷160 mg/d、−5～−2 d，阿糖胞苷160 mg每12 h 1次，−5～−2 d，环磷酰胺1.0 g/d、−5～−2 d）后行自体造血干细胞移植，回输单个核细胞10.8×10^8^/kg、CD34^+^细胞2.1×10^6^/kg。移植后出现Ⅲ级发热伴中性粒细胞减少，予以抗感染治疗，+12 d中性粒细胞植入，+18 d血小板植入。2021年8月随访疗效评估CR，SF 224 µg/L，血常规均恢复正常，未出现HLH复发。

例2，女，54岁，因“颈部淋巴结肿大伴反复发热3个月”于2020年5月入院。血常规示WBC 2.71×10^9^/L，HGB 68 g/L，PLT 33×10^9^/L；骨髓涂片可见噬血细胞；SF 1081 µg/L；EBV-DNA拷贝数1.58×10^7^/L；腹股沟淋巴结活检免疫组化示CD3（+），CD20（−），CD21显示FDC网增多、变形、围绕血管分布，Ki-67 30％～40％（+），CD10（局部+），Bcl-6（局部+），CXCL-13（+），PD-1部分细胞（+），CD4（+）细胞数量多于CD8（+）细胞，CD30散在活化细胞（+），EBER散在（+）；PET/CT示“多发淋巴结肿大伴FDG代谢增高（SUVmax 6.6），脾大伴FDG代谢增高（SUVmax 5.1）”。诊断为“血管免疫母细胞淋巴瘤（AITL）Ⅳ期B组，外周T细胞淋巴瘤非特指型预后指数3分伴HLH”，行CHOPE（环磷酰胺+多柔比星+长春新碱+依托泊苷+泼尼松）、P-Gemox（培门冬酶+吉西他滨+奥沙利铂）各1周期后仍有发热伴噬血现象，sCD25 17 328 ng/L，EBV-DNA拷贝数正常，改以BVED方案（苯达莫司汀+依托泊苷+长春地辛+地塞米松）化疗，首疗程后出现Ⅱ级低蛋白血症、Ⅲ级心力衰竭，超滤脱水14 kg后缓解，2个周期后PET/CT评估达PR，4个周期后增强CT“未见肿大淋巴结”、骨髓未见噬血细胞，SF 2 725 µg/L，sCD25 6 604 ng/L，疗效评估为CR。2020年9月予大剂量依托泊苷行外周血干细胞动员，1个月后再以普乐沙福行外周血干细胞动员、采集，2020年11月复查全身增强CT示CR，12月BEAC方案预处理（卡莫司汀450 mg、−6 d，依托泊苷180 mg/d、−5～−2 d，阿糖胞苷180 mg每12 h 1次、−5～−2 d，环磷酰胺1.2 g/d、−5～−2 d）后进行自体造血干细胞移植，回输单个核细胞10.79×10^8^/kg，CD34^+^细胞4.63×10^6^/kg，期间出现Ⅲ级发热伴中性粒细胞减少，Ⅳ级血小板减少及Ⅱ级γ-谷氨酰转移酶（GGT）升高，予抗感染治疗、重组人血小板生成素（TPO）和保肝治疗后好转，+14 d中性粒细胞植入，+17 d血小板植入。2021年6月随诊复查SF 1 864 µg/L，sCD25 1 364 µg/L，EBV-DNA拷贝数正常。未出现HLH复发。

讨论：本组2例患者的诊疗经过初步显示BVED方案对复发难治非霍奇金淋巴瘤合并HLH有效且毒性可控，对自体造血干细胞采集和自体造血干细胞移植耐受性影响较小，远期疗效尚需继续观察。

